# The association between serum folate and ultrasound - defined hepatic steatosis

**DOI:** 10.1080/07853890.2023.2168042

**Published:** 2023-01-17

**Authors:** Xingxing Chen, Jiajia Lu, Qi Xu, Bin Chen, Lijun Shen

**Affiliations:** Department of Ultrasound, The First People’s Hospital of Xiaoshan District, Hangzhou, P.R. China

**Keywords:** Serum folate, controlled attenuation parameter, hepatic steatosis, NHANES, cross-sectional study

## Abstract

**Purpose:**

It has been discovered that a folate shortage may raise the risk of hepatic steatosis. We investigated the relationship between serum folate and controlled attenuation parameter (CAP) among 3606 participants over from the National Health and Nutrition Examination Survey (NHANES).

**Materials and methods:**

Multivariate logistic regression studies were carried out to calculate the relationship between serum folate and CAP. Additionally, generalized additive models and fitted smoothing curves were carried out.

**Results:**

After adjusting for other variables, we discovered that serum folate had a negative correlation with CAP. Males and whites maintained a negative correlation of serum folate with CAP when subgroup analyses were stratified by sex and race/ethnicity. The relationship between blood folate levels and CAP in whites had an U-shaped curve (inflection point: 34 ng/ml).

**Conclusion:**

According to our study, the majority of Americans, particularly men and whites, had a negative correlation between serum folate and CAP. Among white people, this connection followed an U-shaped pattern. These findings may provide guidance for monitoring serum folate level and controlling oral folate dosage in clinic, so as to prevent liver steatosis more effectively.

**Key Messages**
- The size of the cohort in our study is large, and our findings come from a nationally representative database.
- Our study revealed a negative relationship between serum folate and CAP among most Americans, especially in male and whites, which may provide evidence for medications to treat hepatic steatosis.
- In whites, the association of serum folate with CAP was an U-shaped curve (inflection point: 34 ng/ml). This may provide guidance for monitoring serum folate level and controlling oral folate dosage in clinic, so as to prevent liver steatosis more effectively.

## Introduction

Nonalcoholic fatty liver disease（NAFLD）affects roughly one out of every four Americans and is likely to rise to one out of every three in the next decade [1]. Patients with NAFLD have a higher all-cause death rate than the general population, and it varies by disease stage [2]. In these patients, cardiovascular problems are the most common cause of death, followed by metabolic and liver-related reasons [3]. In NAFLD patients, however, cardiovascular disease is associated with higher levels of steatosis. Increased fat buildup in the form of triglycerides in hepatocytes is referred to as hepatic steatosis (HS) [4]. HS can proceed to cirrhosis and liver failure depending on the many causes and the resulting inflammation and fibrosis [5]. As a result, detecting and quantifying HS in its early phases is critical.

Biopsy, the current gold standard for determining the amount of fat in the liver, has certain drawbacks. Hepatic steatosis is diagnosed and quantified noninvasively using two different but complementing approaches: Biomarkers or imaging, the most common methods are hepatic ultrasonography, controlled attenuation parameter (CAP), computed tomography (CT), as well as magnetic resonance imaging (MRI) [4].

Approaches that restrict cholesterol transport to the liver could theoretically be utilized to prevent or reverse steatosis. Even when body weight is not restored to normal, weight loss, whether accomplished by a self-imposed low-calorie diet or bariatric surgery, cures NAFLD and enhances hepatic insulin sensitivity. Contrarily, weight reduction is challenging to achieve and even more challenging to keep off; just 20% of obese persons are able to do so [6]. As a result, pharmaceutical approaches are being aggressively pursued to reverse hepatic steatosis. Folate is a water-soluble B vitamin that is required for one-carbon transfer reactions such as nucleic acid production, methylation events, and sulfur-amino-acid metabolism. Because the liver is a key storage and processing organ for folate [7], it is critical for maintaining folate homeostasis throughout the body [8]. Folate deficiency is a frequent nutrient shortage that affects persons who have liver illness. The effect of serum folic acid in hepatic steatosis, on the other hand, is unknown. The goal of this study was to use controlled transient elastography to look into the link between serum folate and hepatic steatosis utilizing a sizable, nationally typical population from the 2017–2018 National Health and Nutrition Examination Survey (NHANES).

## Materials and methods

### Statement of ethics

The study received permission from the National Center for Health Statistics Research Ethics Review Board, and each participant completed a consent form.

### Study population

NHANES is a sizable, ongoing, and expertly designed cross-sectional national population survey program in the United States (US), collects information on the general population’s diet and health and uses a stratified, multistage, clustered probability sample design to guarantee national representativeness [9].

A total of 5948 people out of the 9254 that took part in the 2017–2018 NHANES cycle detected hepatic steatosis using the controlled attenuation parameter (CAP). We had 3606 individuals in our research overall after excluding those with lacking serum folate data (*n* = 2342).

### Variables

The exposure factor used in the investigation was serum folate. Using tandem mass spectrometry and isotope-dilution high performance liquid chromatography (LC-MS/MS), serum folate was determined. The experiment is conducted by combining the sample (275 μL serum or whole blood hemolysate) with an internal standard mixture and ammonium for the mate buffer. The outcome variable was CAP, which was assessed by liver ultrasonography transient elastography as a marker of liver fatness. The decibels per meter (dB/m) scale was used to characterize the CAP values. Gender, race/ethnicity, smoking behavior, alcohol consumption in the past year, and the existence of diabetes were all used as factors in our study. The continuous covariates were included in our analysis: age, body mass index(BMI), waist circumference (WC), high density lipoprotein (HDL)- cholesterol, alanine aminotransferase (ALT), aspartate aminotransferase (AST),γ- glutamyl transpeptidase (GGT), total cholesterol, triglyceride, serum albumin, serum creatinine, uric acid, platelet count (PLT), and ferritin. Public access to the detailed data on serum folate, CAP, and variables is provided at http://www.cdc.gov/nchs/nhanes/.

### Statistical analysis

We employed a weighted and variance estimate approach to take into consideration the substantial volatility in our data set. An analysis was conducted using a weighted multivariate logistic regression model to examine the association between serum folate and CAP. We used the weighted χ2 test for categorical variables or the weighted linear regression model for continuous variables to determine the difference between each group. Stratified multivariate regression analysis was used to do the subgroup analysis. Using smooth curve fits and generalized additive models, the nonlinear relationship between serum folate and CAP was also investigated. After nonlinearity was discovered, the inflection point in the relationship between serum folate and CAP was calculated using a recursive technique, and a two-piecewise linear regression model was then used on both sides of the inflection point. The program R (http://www.Rproject.org) and EmpowerStats (http://www.empowerstats.com) were used for all analyses, and a P value <0.05 was deemed statistically significant.

## Results

A total of 3606 people were included in our study. The weighted participant characteristics were divided into four groups according to the quartiles of serum folate (Q1: 1.44–10.6 ng/mL; Q2: 10.7–15.0 ng/mL; Q3: 15.1–22.2 ng/mL; and Q4: 22.3–204.0 ng/mL), as shown in Table 1. With the exception of total cholesterol, AST, and ferritin, there were notable changes in the serum folate quartiles’ baseline characteristics. Participants in the highest quartile of serum folate were more likely to be female, non-Hispanic White and less Mexican American or non-Hispanic Black, and had greater levels of HDL-cholesterol and serum albumin as well as lower levels of BMI, WC, and platelet count as well as CAP.

**Table 1.** Weighted characteristics of the study population based on serum folate quartiles.

Serum folate (ng/mL)	Total	Q1 (1.44–10.6)	Q2 (10.7–15.0)	Q3 (15.1–22.2)	Q4 (22.3–204.0)	*p* Value
Age (years)	41.29 ± 18.54	40.65 ± 16.71	37.62 ± 16.56	40.08 ± 18.62	46.66 ± 20.62	<0.0001
Gender (%)						<0.0001
Male	38.15	40.86	38.48	42.96	30.12	
Female	61.85	59.14	61.52	57.04	69.88	
Race/Ethnicity (%)						<0.0001
Mexican American	10.46	11.44	12.24	11.17	7.10	
Other Hispanic	7.22	5.27	8.57	7.26	7.81	
Non-Hispanic White	59.93	54.53	57.90	59.48	67.54	
Non-Hispanic Black	11.34	17.34	11.78	9.70	6.83	
Other Race	11.05	11.42	9.51	12.39	10.73	
Diabetes (%)						0.0038
Yes	7.96	8.00	7.77	7.24	8.88	
No	92.04	92.00	92.24	92.76	91.11	
Smoked at least 100 cigarettes in life (%)						<0.0001
Yes	39.29	47.19	40.14	35.46	34.17	
No	60.71	52.81	59.86	64.54	65.83	
Number of alcohol drinks a day in past year (%)						<0.0001
Never/none	19.84	16.29	11.06	14.53	20.95	
1–2 drinks	54.44	53.95	53.85	60.88	62.40	
3–4 drinks	17.05	17.94	24.59	16.66	13.12	
≥5 drinks	8.68	11.82	10.50	7.94	3.52	
BMI (Kg/m^2^)	29.12 ± 7.51	30.85 ± 8.51	29.23 ± 7.27	29.08 ± 7.53	27.37 ± 6.13	<0.0001
Waist circumference (cm)	97.80 ± 18.51	101.16 ± 19.24	98.00 ± 18.42	97.41 ± 18.85	94.80 ± 16.93	<0.0001
Laboratory features						
Total cholesterol (mg/dl)	182.75 ± 38.84	183.33 ± 40.46	182.82 ± 39.71	180.63 ± 35.93	184.36 ± 39.24	0.2077
HDL- cholesterol (mg/dl)	53.94 ± 14.36	51.05 ± 14.14	52.97 ± 13.98	54.52 ± 13.82	57.04 ± 14.83	<0.0001
Triglyceride (mg/dl)	131.31 ± 100.47	140.28 ± 105.85	133.87 ± 126.96	123.20 ± 71.17	128.81 ± 92.54	0.0028
AST (IU/L)	21.38 ± 12.64	20.77 ± 14.87	21.90 ± 13.23	21.11 ± 10.63	21.78 ± 11.64	0.1962
ALT (IU/L)	21.34 ± 17.08	20.91 ± 15.34	23.30 ± 21.50	20.29 ± 12.68	21.05 ± 17.90	0.0016
GGT (IU/L)	25.48 ± 30.09	29.40 ± 37.04	27.06 ± 34.31	22.76 ± 23.90	23.11 ± 22.89	<0.0001
Serum albumin (g/L)	41.01 ± 3.21	39.85 ± 3.34	41.18 ± 3.09	41.45 ± 2.98	41.52 ± 3.16	<0.0001
Serum creatinine (mg/dl)	0.83 ± 0.35	0.86 ± 0.49	0.81 ± 0.22	0.83 ± 0.32	0.82 ± 0.33	0.0075
Uric acid (mg/dl)	5.15 ± 1.37	5.25 ± 1.42	5.14 ± 1.46	5.15 ± 1.31	5.05 ± 1.31	0.0281
Platelet count (1000 cells/uL)	251.49 ± 63.84	261.17 ± 69.45	250.71 ± 59.10	248.76 ± 62.10	245.73 ± 63.22	<0.0001
Ferritin (ng/ml)	122.13 ± 144.58	129.86 ± 157.03	115.90 ± 152.80	123.77 ± 131.39	118.78 ± 136.83	0.1965
CAP (dB/m)	255.25 ± 63.35	264.24 ± 64.55	256.94 ± 61.99	253.86 ± 65.92	246.42 ± 59.27	<0.0001

Mean ± SD for continuous variables: the *p*-value was calculated by the weighted linear regression model. (%) for categorical variables: the *p* value was calculated by the weighted chi-square test.

Table 2 displays the outcomes of the multivariate regression analysis. Serum folate had a poor correlation with CAP in the unadjusted model (β= −0.35, 95%CI: −0.55, −0.16, *p* = 0.0004). This adverse correlation persisted in models 2 (β= −0.70, 95%CI: −0.89, −0.52, *p* < 0.0001) and model 3 (β= −0.22, 95%CI: −0.40, −0.04, *p* = 0.0188) after adjusting for covariates. Individuals in the top quartile had a 9.43 dB/m lower CAP than those in the lowest serum folate quartile after categorizing serum folate from a continuous variable to a categorical variable (quartiles).

**Table 2.** The association between serum folate (ng/mL) and controlled attenuation parameter (dB/m).

	Model 1 β (95% CI) *p*-value	Model 2 β (95% CI) *p*-value	Model 3 β (95% CI) *p*-value
Serum folate (ng/mL)	−0.35 (−0.55, −0.16) 0.0004	−0.70 (−0.89, −0.52) <0.0001	−0.22 (−0.40, −0.04) 0.0188
Serum folate categories			
Q1 (1.44–10.6 ng/mL)	Reference	Reference	Reference
Q2 (10.7–15.0 ng/mL)	−7.30 (−13.23, −1.36) 0.0160	−4.35 (−9.85, 1.16) 0.1217	−2.28 (−7.56, 3.00) 0.3968
Q3 (15.1–22.2 ng/mL)	−10.38 (−16.14, −4.61) 0.0004	−11.06 (−16.40, −5.72) <0.0001	−6.72 (−12.02, −1.43) 0.0128
Q4 (22.3–204.0 ng/mL)	−17.81 (−23.66, −11.97) <0.0001	−23.40 (−28.90, −17.90) <0.0001	−9.43 (−14.95, −3.91) 0.0008
Subgroup analysis stratified by sex			
Men	−0.04 (−0.36, 0.28) 0.7898	−0.50 (−0.81, −0.20) 0.0012	−0.40 (−0.68, −0.11) 0.0061
Women	−0.42 (−0.66, −0.18) 0.0007	−0.82 (−1.06, −0.59) <0.0001	−0.09 (−0.33, 0.15) 0.4622
Subgroup analysis stratified by race/ethnicity			
Mexican American	−1.62 (−2.35, −0.89) <0.0001	−1.59 (−2.28, −0.89) <0.0001	−0.45 (−1.31, 0.41) 0.3094
Other Hispanic	−0.53 (−1.33, 0.26) 0.1887	−0.71 (−1.48, 0.06) 0.0719	−0.17 (−0.91, 0.57) 0.6529
Non-Hispanic White	−0.32 (−0.63, −0.01) 0.0441	−0.73 (−1.02, −0.43) <0.0001	−0.22 (−0.49, −0.02) 0.0488
Non-Hispanic Black	−0.22 (−0.69, 0.25) 0.3579	−0.34 (−0.80, 0.11) 0.1412	−0.20 (−0.65, 0.24) 0.3703
Other Race	−0.28 (−0.70, 0.14) 0.1952	−0.54 (−0.95, −0.14) 0.0085	0.02 (−0.53, 0.49) 0.9445

Model 1: no covariates were adjusted.

Model 2: age, sex, and race/ethnicity were adjusted.

Model 3: age, sex, race/ethnicity, body mass index, waist circumference, smoking behavior, alcohol consumption in the past year, the presence of diabetes, high density lipoprotein-cholesterol, alanine aminotransferase, aspartate aminotransferase, γ-glutamyl transpeptidase, total cholesterol, triglyceride, serum albumin, serum creatinine, uric acid, platelet count, and ferritin were adjusted.

In the subgroup analysis stratified by sex and race/ethnicity, the model is not adjusted for sex and race/ethnicity, respectively.

**Figure 1.**
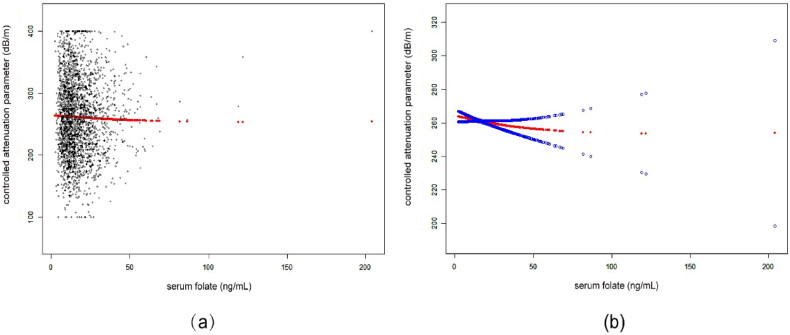
The association between serum folate and controlled attenuation parameter. (a) Each black point represents a sample. (b) Solid rad line represents the smooth curve fit between variables. Blue bands represent the 95% of confidence interval from the fit. Age, sex, race/ethnicity, body mass index, waist circumference, smoking behavior, alcohol consumption in the past year, the presence of diabetes, high density lipoprotein-cholesterol, alanine aminotransferase, aspartate aminotransferase, γ-glutamyl transpeptidase, total cholesterol, triglyceride, serum albumin, serum creatinine, uric acid, platelet count, and ferritin were adjusted.

**Figure 2.**
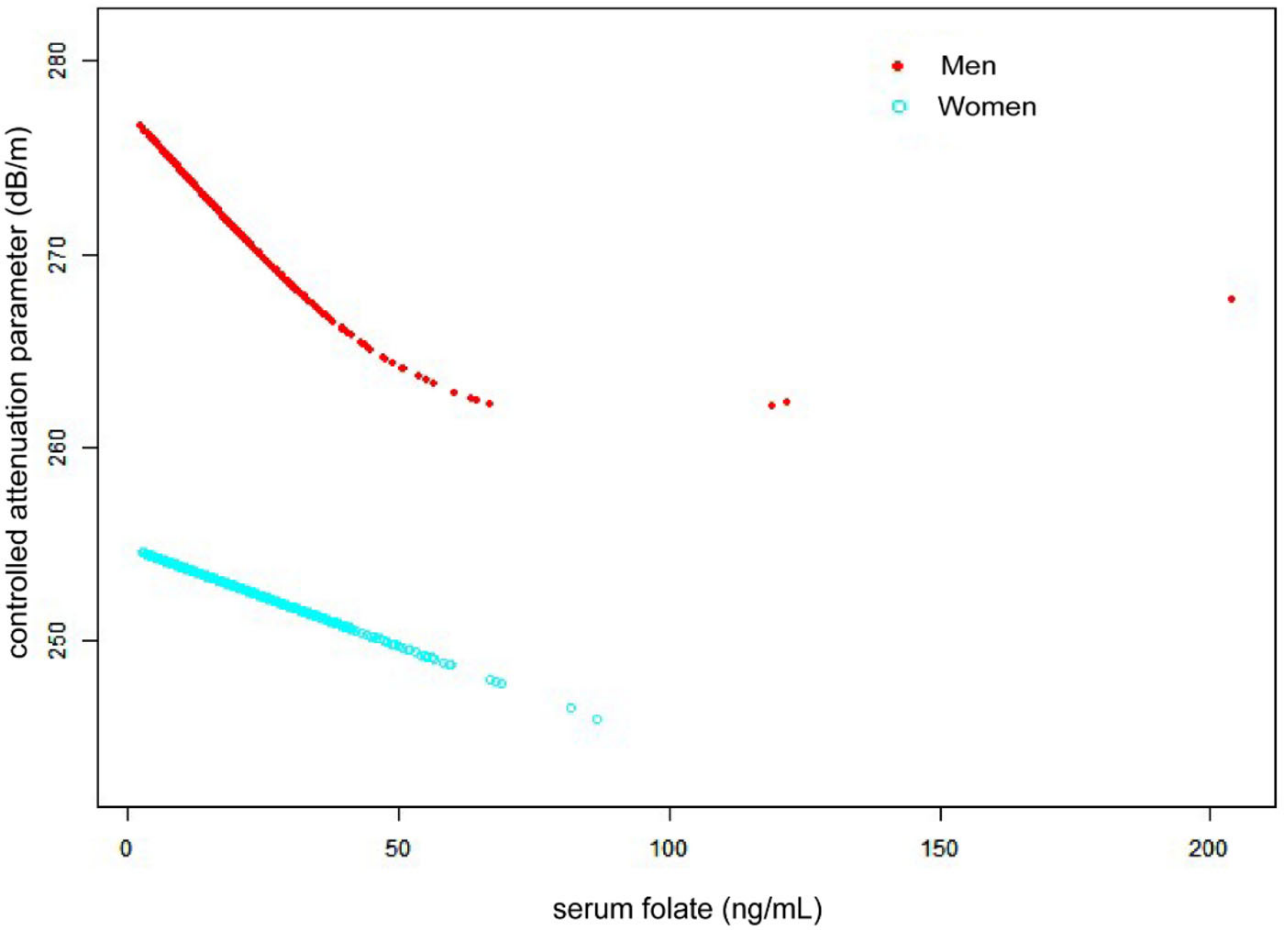
The association between serum folate and controlled attenuation parameter stratified by sex. Age, race/ethnicity, body mass index, waist circumference, smoking behavior, alcohol consumption in the past year, the presence of diabetes, high density lipoprotein-cholesterol, alanine aminotransferase, aspartate aminotransferase, γ-glutamyl transpeptidase, total cholesterol, triglyceride, serum albumin, serum creatinine, uric acid, platelet count, and ferritin were adjusted.

**Figure 3.**
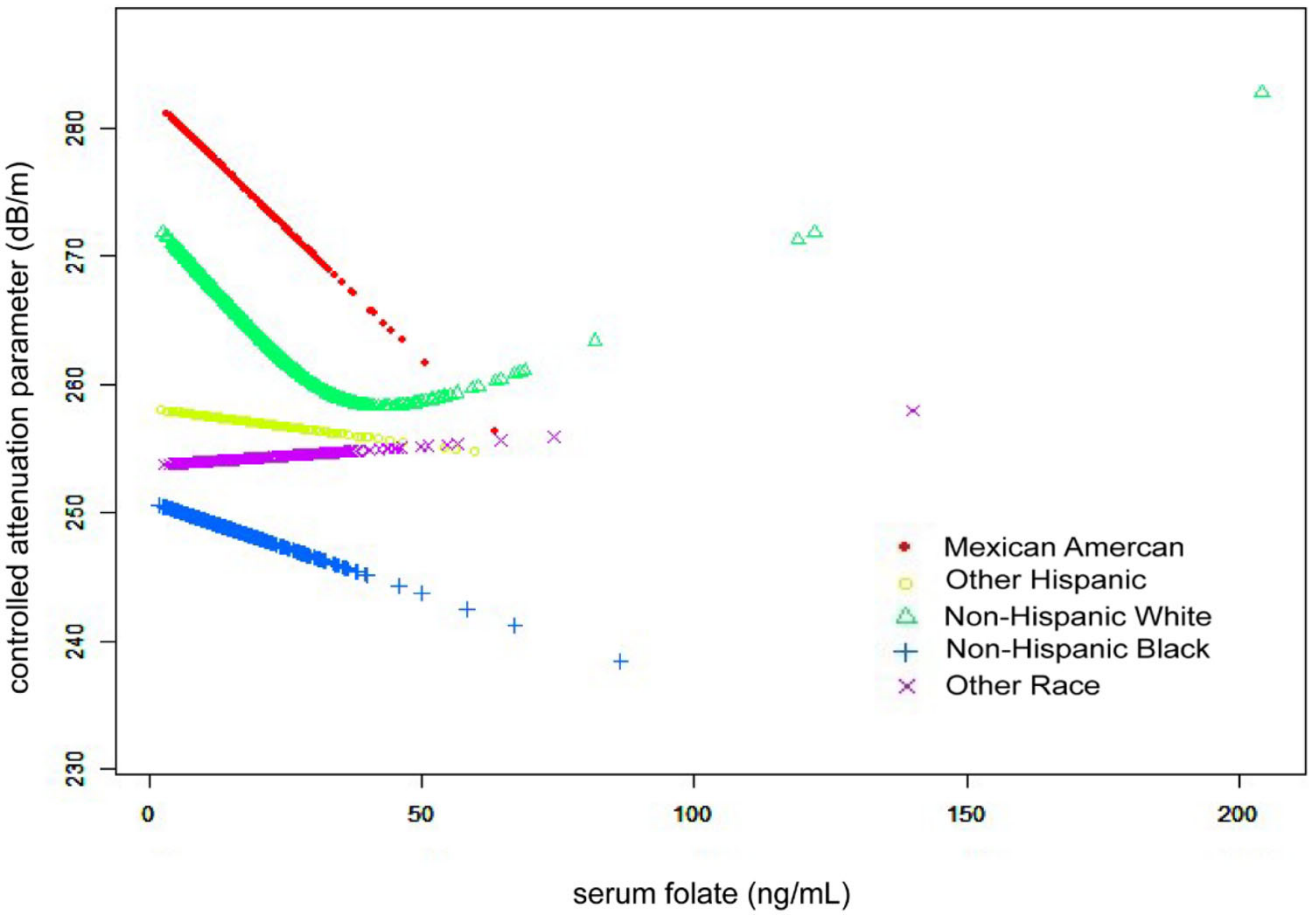
The association between serum folate and controlled attenuation parameter stratified by race/ethnicity. Age, sex, body mass index, waist circumference, smoking behavior, alcohol consumption in the past year, the presence of diabetes, high density lipoprotein-cholesterol, alanine aminotransferase, aspartate aminotransferase, γ-glutamyl transpeptidase, total cholesterol, triglyceride, serum albumin, serum creatinine, uric acid, platelet count, and ferritin were adjusted.

According to subgroup analyses by sex and race/ethnicity, shown in Table 2, the negative connection between serum folate and CAP persisted for both men (β= −0.40, 95%CI: −0.68, −0.11, *p* = 0.0061), and whites (β= −0.22, 95%CI: −0.49, −0.02, *p* = 0.0488). Figures 1–3 depict the smooth curve fits and generalized additive models that were used to describe the nonlinear association between serum folate and CAP. The point of inflection for the U-shaped relationship between serum folate and CAP in whites was found to be 34 ng/mL using a two-piecewise linear regression model (Table 3). For a serum folate <34 ng/mL, every 1 ng/mL upregulation in serum folate was linked to a 0.62 dB/m decrease CAP (95%CI: −1.02, −0.22, *p* = 0.0026); by comparison, for individuals with a serum folate >34 ng/mL, a 1 ng/mL upregulation in serum folate was linked to a 0.30 dB/m greater in CAP (95%CI: −0.11, 0.71, *p* = 0.1533).

**Table 3.** Threshold effect analysis of serum folate on controlled attenuation parameter in non-Hispanic Whites using the two-piecewise linear regression model.

Controlled attenuation parameter	Adjusted β (95% CI), *p* value
Non-Hispanic White	
Fitting by the standard linear model	−0.17 (−0.40, 0.07) 0.1656
Fitting by the two-piecewise linear model	
Inflection point	34
Serum folate <34 (ng/mL)	−0.62 (−1.02, −0.22) 0.0026
Serum folate >34 (ng/mL)	0.30 (−0.11, 0.71) 0.1533
Log likelihood ratio	0.006

Age, sex, body mass index, waist circumference, smoking behavior, alcohol consumption in the past year, the presence of diabetes, high density lipoprotein-cholesterol, alanine aminotransferase, aspartate aminotransferase, γ-glutamyl transpeptidase, total cholesterol, triglyceride, serum albumin, serum creatinine, uric acid, platelet count, and ferritin were adjusted.

## Discussion

In the 2017–2018 NHANES project, transient elastography was utilized to evaluate hepatic steatosis, providing the first representative observations of the biggest sample size in the United States for transient elastography CAP. According to multivariate logistic regression analysis, higher serum folate levels were linked to reduced CAP in our sample, with the link being stronger in men. Our findings are in line with other research that implicates a folate shortage in the emergence of various small sample groups or animal models of liver injury. A research in obese adults found that patients with confirmed severe NAFLD liver biopsies had lower serum folate concentrations than those with normal livers or minor liver abnormalities [10]. And Halsted et al. found minor steatosis in two of six alcohol-fed animals with adequate folate intake, but steatonecrosis in five of six ethanol-fed folate-deficient micropigs [11]. Our research adds to and verifies this link in a wide group of people, providing evidence for medications to treat hepatic steatosis.

Hepatic steatosis risk is elevated in cases of folate insufficiency, possibly because a lack of folate is linked to increased expression of lipid biosynthesis genes, which causes lipid metabolism in the liver to be disrupted [12]. In addition, some studies suggest that in folate-deficient animals, hepatic lipid transport is hindered, which promotes hepatic fat buildup [13–15].

Furthermore, we discovered a nonlinear connection between serum folate and CAP in whites in a subgroup analysis, with an inflection point at 34 ng/ml. To our knowledge, this study may be the first to demonstrate a connection between serum folate and CAP in whites. Whites had much greater serum folate levels than blacks and Mexican Americans, according to the research [16]. The identified disparities in risk variables by race might be explained by variations in genetic risk factors, obesity prevalence, alcohol use, and other factors. Additional prospective studies with sizable sample sizes were required to elucidate the relationship between blood folate and CAP in white people. The size of the cohort in our study strengthens the findings since the NHANES is intended to yield nationally representative estimates. Nevertheless, our study still had a number of restrictions or flaws. First, hepatic steatosis was defined by transient elastography using the CAP values rather than pathologically confirmed by biopsy, which may introduce bias in assessing the extent of hepatic steatosis. Second, confounding factors that were self-reported might be subject to self-report bias. Third, conclusions in this study are limited to association rather than causality because of the cross-sectional design.

## Conclusions

Most Americans have a negative connection between serum folate and CAP, according to our research. This connection among whites followed a U-shaped curve, and the lowest value of CAP occurred when the serum folate level reached the inflection point. These findings may provide guidance for monitoring serum folate level and controlling oral folate dosage in clinic, so as to prevent liver steatosis more effectively. Attention to folate research may lead to the discovery of pharmacologic methods to reverse hepatic steatosis.

## Data Availability

The data that support the findings of this study are available from NHANES and don’t require any permissions. Data sharing is not applicable to this article as no new data were created or analyzed in this study.
